# Understanding family functioning and social support in unremitting schizophrenia: A study in India

**DOI:** 10.4103/0019-5545.64593

**Published:** 2010

**Authors:** Neena S. Sawant, Kamal S. Jethwani

**Affiliations:** Department of Psychiatry, KEM Hospital, Parel, Mumbai - 400 012, India

**Keywords:** Family functioning, schizophrenia, social support

## Abstract

**Context:**

This study aimed to clarify the difference in the perception of family functioning and social support by the schizophrenic patients and their principal caretakers, and whether the social support is related to healthy family functioning.

**Setting and Design:**

The study was set in the psychiatric outpatient department of a tertiary care hospital and data was collected by means of a semi-structured interview.

**Materials and Methods:**

Fifty unremitting schizophrenics diagnosed by diagnostic and statistical manual (DSM)-IV criteria and their family members were interviewed. Family functioning was assessed by the family assessment device (FAD) and the social support was assessed by the multidimensional scale of perceived social support (MSPSS).

**Statistical Analysis:**

Group differences were analyzed using unpaired ‘*t*’ test for comparison of FAD and MSPSS means and subscale scores. Pearson’s correlation coefficient was used to find the direction and magnitude of association between the various dimensions (subscales) of FAD and the social support from family.

**Results:**

Schizophrenic patients had more difficulty on problem solving as compared to their relatives, while no significant differences were noted on the other dimensions of FAD in the two groups. Also, schizophrenics perceived more social support from friends than from their families. All the dimensions of the family functioning correlated to the social support perceived from the family in the schizophrenic patients.

**Conclusions:**

Our study highlights the need to study the issues of perception of family functioning and social support so as to improve the prognosis in a disabling disorder like schizophrenia. Providing better social support and understanding the family functioning will result in strengthening the family as a unit, so as to provide better care to the patient.

## INTRODUCTION

Family is a basic unit that is responsible in preserving the integrity of individuals, who form the unit. Families extend emotional, social, and economic support to their members. A high functioning family helps in maintaining the dimensions of communication, emotional and behavior control, and also helps in problem solving and coping behaviors of its members. An illness like schizophrenia is serious and disabling and causes an emotional and financial brunt on the supporting family members.

In the last three decades, increasing research has been done on the role of the family in the course of schizophrenia. Importance has been given to the family environment as a contributing factor to the relapse or rehabilitation of the patient.[1,2]

This laid the ground for the concept of expressed emotions (EE), which refers to the attitudes of the family members toward the patient and has wide implications in the course and prognosis of the disorder.[3] This has resulted in family interventions aimed at reducing the negative impacts of the caregivers on the patient’s rehabilitation and recovery.[4,5]

Thus, the family is an important factor which affects the patient’s mental well-being and outcome. However, the family may itself be burdened by the enormous hardships created by the schizophrenic patient. A comprehensive and empathic understanding of the family members on various dimensions would throw more light in determining the behavioral patterns existing in the family and development of newer treatment techniques.

Hence, instead of focusing on a single dimension of EE, families should be assessed on the various dimensions as proposed by the McMaster model of family functioning to give a more holistic view.[6]

In India, several studies have focused on the burden of schizophrenia on families and social support, but there is a dearth of literature on studying the various dimensions of the family functioning as per the McMaster model.

Schizophrenics have several needs regarding social support, welfare benefits, education about the illness, and psychiatric distress. Also, they have deteriorating relations and careers resulting in increased isolation and loss of social support.[4]

A patient’s support system may come from several sources apart from family such as friends, residential or daycare providers, shelter operators, roommates, and others. It is necessary to know from whom the patient perceives social support. This will ensure proper social support, encouragement, and treatment. Research has shown that a family with a schizophrenic patient does suffer from network contraction and condensation, which in turn, increases the vulnerability of the family to stressors due to lack of social support.[7]

The Indian patient has an advantage of being in a family system which believes in extending social support. Most of the families are joint or nuclear as a result of which there is a decreased burden of emotional, social, and economic factors on the primary support group. Antronucci and Israel (1986), and Cohen and McKay (1984) have found that social support functions against pathology by:

- Helping people mobilize their psychological resources so as to enable them to deal with emotional problems;
- Providing a helping hand in sharing tasks;
- Giving emotional help; and
- Helping to deal with stressful life situations.[8,9]

Family functioning and social support are important factors in psychiatric treatment. The perception of the psychiatric patients regarding their family may be distorted due to their disorders. Hence, an understanding of their perceptions of family functioning is important as it predicts the clinical outcomes.[10,11] It is equally important to know how each family member perceives family functioning, as studies have found a discrepancy in the perceptions of family functioning between patients and their family members.[10,12,13]

This study was undertaken with the following objectives:

1. To clarify the difference in the perception of family functioning by schizophrenics and their immediate family members, that is, principal caretaker.
2. To study the differences in the perceived social support in both the groups.
3. To assess how the various dimensions of the family functioning relate to the social support from family in the schizophrenic group.

These objectives would have clinical implications when evaluating family functioning in psychiatric practices.

## MATERIALS AND METHODS

### Subjects

This study was approved by the institutional ethics committee. It was conducted in the psychiatric outpatient department (OPD) of a tertiary care hospital.

Inclusion criteria for the patient group were:

- Patients who met the DSM-IV criteria for schizophrenia.[14]
- Patients who did not live independently.
- Patients who were on regular treatment and follow up from the psychiatric OPD and had unremitting schizophrenia (i.e., still had positive or negative symptoms, bizarre behaviors, lack of personal hygiene, etc, despite being on treatment.)

Inclusion criteria for the principal caretaker group were:

- They were the immediate family members or principal caretaker of the patient.
- They lived with the patient.

Exclusion criteria for both the groups were:

- Any comorbid psychiatric or medical illness in the last six months.
- Schizophrenics who were well maintained on treatment.
- Subjects below 18 years of age.

Eligible patients and their relatives, who visited the psychiatric OPD between January 2005 and June 2005, received an explanation of the study and its implications and written informed consent was obtained for participation in the study from the patients and their family members. A *proforma* was designed in the form of a semi-structured interview to collect information on demographic variables, duration of illness, and treatment taking behavior.

A total of 62 patients and their family members were screened. Of these, 50 patients and their family members satisfying the above criteria were recruited for the study, which thus formed the two groups.

### Instrument

#### Family assessment device

The FAD is a standardized measure for assessing family functioning. It is a 60-item self-reporting questionnaire developed by Epstein (1983) based on the McMaster model of family functioning.[6] The FAD gives a total score and seven subscale scores. Each FAD item is scored on a 4-point scale (including reverse items) with higher scores indicating poorer or worse family functioning. The FAD is designed such that the families can be understood along the following seven dimensions:

- Problem solving, which concerns the families’ ability to proceed through seven steps of problem identification to problem resolution in both instrumental and affective areas;
- Communication, which refers to the effectiveness and extent of the family’s style of communication;
- Roles, which are the recurrent patterns of behavior necessary to satisfy the instrumental and affective needs of the family members;
- Affective responsiveness, which measures the capacity of family members in giving response with the appropriate quality and quantity of feelings to a wide range of stimuli like love, happiness, anger, and sadness;
- Affective involvement, which refers to the amount of interest, care, and concern family members invest in each other;
- Behavioral control, which defines the family’s style of maintaining discipline and standards of behavior, for example, flexible or rigid; and
- General functioning, which assesses the overall impact of the family on the daily functioning of the respondent.

Based on this model, the McMaster family assessment device (FAD) was developed and a number of studies found it reliable and valid in assessment of a wide range of families in psychiatric, nonclinical, and other medical samples.[15,16]

#### Assessment of social support

MSPSS developed by G. D. Zimet was used to measure the social support as perceived by the individual.[17] It is a twelve-item questionnaire which evaluates social support from family members, friends, and significant others, and quantifies the degree to which respondents perceive social support from each of these sources. MSPSS gives a total score and three subscale scores.

A translation expert translated the FAD and the MSPSS into two Indian languages, Hindi and Marathi, which was then checked by two other psychiatrists from the department of psychiatry for accuracy and relevance before it was administered to the patients.

### Statistical analysis

Group differences were analyzed using unpaired ‘*t*’ test for comparison of FAD and MSPSS mean scores between the patients and their family members.

Pearson’s correlation coefficient was used to find the direction and magnitude of association between the various dimensions of FAD and the social support from family. All statistical analyses were performed with SPSS 11 for Windows with a significant level set at *P*<0.05 (two tailed).

## RESULTS

### Characteristics of the sample

The demographic structure of the sample showed the mean age of the patient group as 34 ± 12.61 years. The study population consisted of 35 (70%) men and 15 (30%) women. Thirty five of them (70%) had secondary or higher education, whereas five (10%) were illiterates. Twenty-one subjects (42%) were unmarried and 23 (46%) were unemployed. Fourty subjects (80%) stayed in a nuclear family. The minimum duration of illness was 1 year, and maximum was 20 years. The mean duration of illness was 4.28 years.

The mean age of the relatives group was 39.9 ± 12.37 years. Of the total, 68% were women and 16% were men. Majority of the primary caretakers were parents or spouses. Twenty one (42%) of them were illiterates whereas 24 (48%) had secondary and higher education. Fourty (80%) were married and only eight (16%) were unmarried. Twenty four (48%) of them were employed, while the rest were unemployed. Majority of the subjects (94%) were hindus.

#### Comparison of family assessment device scores

Table 1 shows the FAD mean scores of patients and their family members. A significant difference in problem solving was seen between the groups, with the schizophrenic patients having more difficulty as compared to their relatives. However, no statistically significant difference was seen on the other dimensions of FAD.

**Table 1:** Family assessment device

FAD subscales	Patient group		Relative group		*t* test	*P* value
	Mean	SD	Mean	SD		
Roles	30.02	4.779	28.82	3.462	1.438	0.1536
Problem solving	13.92	3.4	12.24	2.52	2.805	0.0068**
Communication	22.1	3.34	21.12	5.14	1.13	0.2614
Affective involvement	13.7	2.94	13.5	13.5	0.353	0.72
Behavior control	23.92	2.35	23.72	23.72	0.326	0.74
Affective responsiveness	13.2	2.8	14.3	2.9	1.93	0.056
General functioning	30	4.49	28.6	28.6	1.45	0.148

* *P*<0.05;

** *P*<0.01

#### Comparison of multidimensional scale of perceived social support scores

Table 2 shows the MSPSS mean scores of patients and their family members. A significant difference was seen with the schizophrenic patients perceiving more social support from friends as compared to their relatives. No significant difference was seen in both the groups in perceiving social support from family and from significant others.

**Table 2:** Social support by MSPSS

MSPSS subscales	Patient group		Relative group		*t* test	*P* values
	Mean	SD	Mean	SD		
Support from family	12.1	5.68	11.3	4.164	0.7915	0.43
Support from friends	29.2	9.2	25.2	9.1	2.141	0.034*
Support from significant others	2.98	1.23	2.7	1.2	0.981	0.328

* *P*<0.05; MSPSS: Multidimensional scale of perceived social support

#### Comparison between social support and FAD scores of patients

Table 3 shows the Pearson’s correlation coefficient (r) of FAD scores and the MSPSS scores of the social support from family of the patients. All dimensions of the family functioning correlated with the perceived social support from the family in the schizophrenic sample.

**Table 3:** Correlation between family assessment device and social support subscales of schizophrenic group

FAD	MSPSS social support from friends	
	*r*	*P*
Roles	–20.3446	0.01*
Problem solving	0.34	0.013*
Communication	–20.29	0.03*
Affective involvement	–20.29	0.03*
Behavior control	–20.37	0.006**
Affective responsiveness	–20.47	0.002**
General functioning	–20.38	0.006**

* *P*<0.05;

** *P*<0.01; MSPSS: Multidimensional scale of perceived social support

The correlations of behavior control, affective responsiveness, and general functioning were highly significant. Thus providing support to the notion that despite the level of schizophrenia, if the social support perceived from the family is higher, it would help in the better functioning of the family.

## DISCUSSION

The characteristics of the patient sample did reveal a male preponderance, while the caretakers were predominantly mothers or wives of the patients. Majority of the schizophrenics had secondary education and above as compared to their caretakers, who were illiterates. The schizophrenic patients were also found to have achieved education and only five of them (10%) were illiterates.

Despite the Indian culture where families stay in joint or extended family structure (where more than two generations live together under one roof, in a patriarchal system of family functioning, with the eldest male being the head of the house), our sample had 80% staying in nuclear families, which would then have implications on the prognosis of the illness.

This study investigated the family functioning of the unremitting schizophrenics and compared the different dimensions with the caretakers of the schizophrenics. The schizophrenics were found to have a poorer problem solving ability as compared to their relatives. Similar findings were found by Koyama *et al*.[13] Addington *et al*., found a positive correlation between neurocognitive functioning and interpersonal problem solving skills in patients with schizophrenia.[18] Cognitive deficits of psychotic patients could also lead to their negative perception about the problem solving functioning of their family. On the other dimensions of family functioning also, the schizophrenic group perceived a decreased ability of the family to perform behaviors to satisfy the instrumental and affective needs of the other family members (roles), transmit clear and directive verbal messages (communication), show interest and care to one another (affective involvement), and maintain the standard of discipline as compared to their caretakers, though these were not found to be statistically significant. However, the caretakers perceived more dysfunction than the patients in affective responses of appropriate quality, though it just missed the test for significance.

Schizophrenics, owing to the illness, had a difficulty in their family functioning as the patient’s perception of the family functioning could reflect the characteristics of their disorders. Studies have noted a discrepancy on family functioning between patients and family members depending on the patients’ diagnosis of schizophrenia, depression, or bipolar disorder.[13]

The extent of perceived social support received from family or from significant others did not reveal any significant differences in both the groups. However, the schizophrenic patients did perceive more support from their friends as compared to their caretakers. This could be due to the social structure where neighbors and family friends almost become a part of the family due to increased social interactions during cultural activities and festivities. The family which is burdened by the mental illness is in need of emotional, financial, and instrumental support to ensure its healthy functioning. Researchers have found that a family with a schizophrenic member is at a risk of network contraction and condensation, and are often dissatisfied with the social support obtained,[1,7] Despite the illness, the patients in our study found more support from their friends, which is in contrast to the above findings.

Social support was also found to be strongly correlated with the functioning of the families in the schizophrenics. Social support provided by the relatives strongly related to all the dimensions of family functioning. Our study findings are in keeping with those of Yu-Kit Sun and Cheung.[19] Thus, strengthening the family functioning would involve the development of informal supportive networks for the families and to expand the natural social networks. The patients and their families need support and comprehensive family intervention.

The supportive model of multiple family group therapy is also a possible mode of intervention.[20] From the Indian perspective, social support is very much a part of the Indian culture as family, friends, and others often extend emotional and economic support in times of crisis and illness. There is a good networking among people in India who often stand in times of need. Hence, one would not expect much difference from the patients or the caretakers on the perceived social support. However, an illness like schizophrenia is also bound to affect the family in causing an emotional and economic burden as a result of which the primary support group can get a burnout in dealing with their symptoms of schizophrenia. All this would have its impact on the extent of social support offered and the other dimensions of family functioning.

## CONCLUSIONS

The present study highlights that the perceptions of the schizophrenic patients and those of their family members were almost at the same level, except for problem solving. Social support from friends was perceived more by the schizophrenic patients and all the dimensions of the family functioning correlated to the social support from family in the patient group. This shows that it is important to assess family functioning as perceived by the schizophrenic patients as the perceptions of the family environment of a schizophrenic patient often predict his or her relapse and/or re-hospitalization.[10]

The study has some limitations. The sample size was small and we did not employ a nonclinical control, or a comparison with remitting schizophrenics and their caretakers. Comparison between parents and spouses among the caretakers would also provide additional insight into the discrepancy of perception among the family members as well as the patient population. Larger controlled studies would definitely throw more light on the above issues.
